# Adaptation to Blue Light in Marine *Synechococcus* Requires MpeU, an Enzyme with Similarity to Phycoerythrobilin Lyase Isomerases

**DOI:** 10.3389/fmicb.2017.00243

**Published:** 2017-02-21

**Authors:** Rania M. Mahmoud, Joseph E. Sanfilippo, Adam A. Nguyen, Johann A. Strnat, Frédéric Partensky, Laurence Garczarek, Nabil Abo El Kassem, David M. Kehoe, Wendy M. Schluchter

**Affiliations:** ^1^Department of Biology, Indiana University, BloomingtonIN, USA; ^2^Department of Botany, Faculty of Science, University of FayoumFayoum, Egypt; ^3^Department of Biological Sciences, University of New Orleans, New OrleansLA, USA; ^4^Department of Chemistry, University of New Orleans, New OrleansLA, USA; ^5^CNRS, Sorbonne Universités, Université Pierre et Marie Curie University Paris 06, UMR 7144Roscoff, France; ^6^Indiana Molecular Biology Institute, Indiana University, BloomingtonIN, USA

**Keywords:** blue light, lyase isomerase, phycobilin, phycourobilin, phycoerythrin, marine cyanobacteria, phycobilisome, marine *Synechococcus*

## Abstract

Marine *Synechococcus* has successfully adapted to environments with different light colors, which likely contributes to this genus being the second most abundant group of microorganisms worldwide. Populations of *Synechococcus* that grow in deep, blue ocean waters contain large amounts of the blue-light absorbing chromophore phycourobilin (PUB) in their light harvesting complexes (phycobilisomes). Here, we show that all *Synechococcus* strains adapted to blue light possess a gene called *mpeU*. MpeU is structurally similar to phycobilin lyases, enzymes that ligate chromophores to phycobiliproteins. Interruption of *mpeU* caused a reduction in PUB content, impaired phycobilisome assembly and reduced growth rate more strongly in blue than green light. When *mpeU* was reintroduced in the *mpeU* mutant background, the *mpeU-*less phenotype was complemented in terms of PUB content and phycobilisome content. Fluorescence spectra of *mpeU* mutant cells and purified phycobilisomes revealed red-shifted phycoerythrin emission peaks, likely indicating a defect in chromophore ligation to phycoerythrin-I (PE-I) or phycoerythrin-II (PE-II). Our results suggest that MpeU is a lyase-isomerase that attaches a phycoerythrobilin to a PEI or PEII subunit and isomerizes it to PUB. MpeU is therefore an important determinant in adaptation of *Synechococcus* spp. to capture photons in blue light environments throughout the world’s oceans.

## Introduction

With a global abundance of approximately 7 × 10^26^ cells, *Synechococcus* is the second most abundant phytoplanktonic group in the world’s oceans and contributes to approximately 16% of global primary production (Flombaum et al., 2013). The ecological success of these picocyanobacteria likely relies in part on their wide diversity of photosynthetic pigments (Six et al., 2007), which allows for adaptation to different depths in the water column and different oceanic regimes exhibiting various spectral properties (Ong et al., 1984; Farrant et al., 2016; Xia et al., 2017). Most cyanobacteria harvest light using phycobilisomes (PBS), which are large soluble complexes associated with the thylakoid membranes (Ong and Glazer, 1991; Arteni et al., 2009). PBS are composed primarily of phycobiliproteins forming a central core and rods that radiate out from the core. In marine *Synechococcus*, PBS rods may contain three types of phycobiliproteins: phycocyanin (PC), phycoerythrin I (PEI), and/or phycoerythrin II (PEII). Phycobiliproteins are composed of α and β heterodimers, which pack into donut-shaped trimers (αβ)_3_ connected to each other by linker polypeptides (Glazer, 1989; Ong and Glazer, 1991; Six et al., 2007). Each α and β subunit contains open-chain tetrapyrrole chromophores, known as phycobilins, which are ligated to specific cysteine residues (Glazer, 1989). Marine *Synechococcus* phycobiliproteins can contain three different types of isomeric chromophores, which absorb different light wavelengths: phycocyanobilin (PCB) absorbs red light, phycoerythrobilin (PEB) absorbs green light, and phycourobilin (PUB) absorbs blue light (Six et al., 2007).

Each phycobilin bound to α or β subunits has specific spectral properties due to the lengths of their conjugated double bond systems and the stretched orientation of the chromophore through its interaction with the phycobiliprotein and with linker polypeptides (Glazer, 1989). Every phycobilin is bound to conserved cysteine residues via a thioether linkage, a ligation catalyzed by enzymes called phycobilin lyases (Fairchild et al., 1992; Scheer and Zhao, 2008). There are three distinct families of phycobilin lyases: the CpcE/F, the CpcT, and the CpcS/U families (Schluchter et al., 2010; Bretaudeau et al., 2013). Enzymes which belong to the CpcE/F family were studied first (Zhou et al., 1992; Fairchild and Glazer, 1994). They are predicted to form structures that are primarily alpha helical (Shukla et al., 2012), to contain HEAT-repeat motifs (Andrade et al., 2001; Marcotrigiano et al., 2001; Takano and Gusella, 2002; Morimoto et al., 2003), and some members can isomerize the phycobilins during the attachment process (Storf et al., 2001; Blot et al., 2009; Shukla et al., 2012). Members of both CpcT and CpcS/U families of phycobilin lyases form beta barrel structures (Kronfel et al., 2013; Overkamp et al., 2014; Zhou et al., 2014) and are active as homo or heterodimers. CpcS is hypothesized to have evolved first (Biswas et al., 2011) because it can bind to more than one phycobiliprotein subunit. All of the PCB lyases necessary to bind chromophores on allophycocyanin and phycocyanin have been biochemically characterized (Zhou et al., 1992; Fairchild and Glazer, 1994; Zhao et al., 2005, 2006, 2007a,b; Shen et al., 2006, 2008; Saunée et al., 2008; Scheer and Zhao, 2008; Biswas et al., 2010), while the function of only few of the lyases acting on phycoerythrin has been determined to date (Wiethaus et al., 2010; Biswas et al., 2011; Shukla et al., 2012).

Although some marine *Synechococcus* strains have PBS rods constituted only of PC, most *Synechococcus* strains also contain PEI and/or PEII (Swanson et al., 1991; Six et al., 2007). Based on their relative PUB and PEB content, as assessed by the PUB:PEB fluorescence excitation ratio (hereafter Ex_495_:Ex_545_) of whole cells, strains with PEI and PEII have been classified into four pigment types: 3a (low PUB), 3b (medium PUB), 3c (high PUB), and 3d (variable PUB) (Six et al., 2007; Humily et al., 2013). The latter strains perform a process called type IV chromatic acclimation (CA4), during which cells change their PUB:PEB ratio over the course of about six generations to match their absorption properties to the predominant light color, i.e., either blue light (BL) or green light (GL) (Palenik, 2001; Everroad et al., 2006; Shukla et al., 2012). Three chromophore sites were shown to change when cells were shifted between BL and GL: one site on CpeA is PEB in GL and PUB in BL (Cys-139) and two sites on MpeA are PEB in GL and PUB in BL (Cys-83 and Cys-140) (Shukla et al., 2012). Comparative genomic analysis has shown that this ability is conferred by a specific genomic island, called the CA4 island (Humily et al., 2013; Sanfilippo et al., 2016).

PCB and PEB are formed from heme by heme oxygenase and ferredoxin-dependent bilin reductases (e.g., PcyA and PebA/B, respectively) (Frankenberg et al., 2001). Similarly, in the moss *Physcomitrella patens* PUB is synthesized directly by the bilin reductase PubS (Chen et al., 2012). However, no *pubS* homolog has been detected in cyanobacterial genomes sequenced to date. Instead, cyanobacterial PUB is formed by isomerization of PEB during its attachment to a phycobiliprotein, a function performed by bifunctional phycobilin lyases (Blot et al., 2009; Shukla et al., 2012). Thus far, the only lyases that have been shown to have this PEB lyase-isomerase activity are RpcG (Blot et al., 2009) and MpeZ (Shukla et al., 2012), and both proteins are members of the CpcE/F family of lyases. During CA4, it is hypothesized that when GL-acclimated cells sense BL (or vice versa), a new set of lyases (or lyase-isomerases) is transcribed or activated to synthesize PEI and PEII α-subunits with a chromophorylation better matching the new ambient light color and former PBS are progressively replaced by this new set of BL-acclimated PBS (Everroad et al., 2006; Shukla et al., 2012; Humily et al., 2013; Sanfilippo et al., 2016).

Here, we explore the function of MpeU (Wilbanks and Glazer, 1993) another member of the CpcE/F family that is specific to *Synechococcus* strains exhibiting pigment types 3b, 3c, and 3d. We used reverse genetics to interrupt *mpeU* in the model 3d strain *Synechococcus* sp. RS9916. We determined that MpeU is required for high PUB content and that in its absence, mutant PBS are not assembled properly.

## Materials and Methods

### Strain and Culture Conditions

*Synechococcus* sp. RS9916 (hereafter 9916), isolated from the Red Sea Gulf of Aqaba at a 10 m depth (Fuller et al., 2003), was obtained from the Roscoff Culture Collection (strain no. RCC555^1^). *Synechococcus* cultures were grown in PCRS11-Red Sea medium using a final concentration of 1 mM Hepes-NaOH, 8 μM Na_2_-EDTA/FeCl_3_, 50 μM NaPO_4_, 400 μM (NH_4_)_2_SO_4_, 1 μg/L cyanocobalamin and Gaffron+Se^2^. Media was sterilized using a 0.22 μm filter. Cells were grown at 24 ± 1°C under continuous light illumination using Chroma75 fluorescent bulbs 40 W (General Electric). Photon flux was measured with a Li-Cor LI-250 light meter. To generate BL and GL, filters (LE716 Mikkel Blue and LE738 Jas Green; LEE Filters) were used.

### Comparative Genomics and Phylogenetic Analyses

The 54 marine *Synechococcus* or *Cyanobium* genomes used here for comparative genomics were either retrieved from GenBank or assembled *de novo* as previously described (Humily et al., 2013; Farrant et al., 2015) after sequencing at the Genoscope (Evry, France) or at the Center for Genome Research (Liverpool, UK). After a preliminary automatic structural and functional annotation using the Manatee pipeline^3^, orthologous coding sequences were clustered using orthoMCL^4^, then included into the Cyanorak v2 information system^5^ to manually refine the annotation of genes potentially involved in phycobilisome biosynthesis. Unpublished *mpeU* sequences have been submitted to GenBank under accession numbers KY347703–KY347720.

Maximum likelihood trees were inferred using PHYML v3.0 – 20120412 (Guindon and Gascuel, 2003) with the LG+G+I substitution model for MpeU and HKY+I+G for the *petB* gene. Confidence of branch points was determined by performing bootstrap analyses including 1000 replicate data sets. Phylogenetic trees were edited using the Archaeopteryx v0.9901 beta program (Han and Zmasek, 2009). The single MpeU tree was drawn using iTOL^6^ (Letunic and Bork, 2007) and tree comparison was made using the dendextend R package (Galili, 2015).

### Cloning and Construction for the *mpeU* Interruption

Primers and plasmids used in this study are listed in Supplementary Tables S1 and S2, respectively. Int-BamHI-mpeU-for and Int-BamHI-mpeU-rev were used to amplify 485 base pair insert, and this fragment was cloned into the BamHI site of pMUT100 to generate pJASmpeU. The cloned junction was sequenced. pMUT100 is a suicide vector in marine *Synechococcus* conferring resistance to kanamycin and was used to interrupt *mpeU* in 9916 through homologous recombination as described (Brahamsha, 1996; Shukla et al., 2012). Three independent colonies were picked from plates and tested by PCR amplification and Southern blot analysis to confirm the interruption. The transformed mutant lines were grown in PCRS11 media with 50 μg/mL kanamycin. pJS1mpeU was made to express MpeU in the *mpeU* mutant using Comp-BamHI-mpeU-for and Comp-EagI-mpeU-rev. The upstream region of *mpeU* containing the promoter and the *mpeU* gene was cloned into BamHI and EagI sites of pJS1. pJS1 is an autonomously replicating plasmid in marine *Synechococcus* that confers spectinomycin resistance (Sanfilippo et al., 2016) that was originally derived from pRL153 (Brahamsha, 1996). All the transformed lines for complementation were grown in PCRS11 media with 20 μg/mL spectinomycin.

### Whole Cell Absorbance and Fluorescence Spectroscopy

Whole cell absorbance spectroscopy and optical density measurements were performed using a Beckman DU640B spectrophotometer. Data shown are an average of three independent replicates. A Synergy-Mx plate reader (Bio Tek) was used to measure fluorescence excitation and emission.

### Phycobilisome Preparation

Phycobilisomes were prepared with some modification of Gantt et al. (1979). The entire process for PBS preparation was done at room temperature. Cells were collected by centrifugation in mid log phase and resuspended in 0.65 M phosphate buffer (pH 7.5). Then cells were broken using a French Press at 9000 psi and PBS were purified as described (Shukla et al., 2012).

## Results

### *mpeU* is Specific to *Synechococcus* Strains with High PUB Content

**FIGURE 1:**
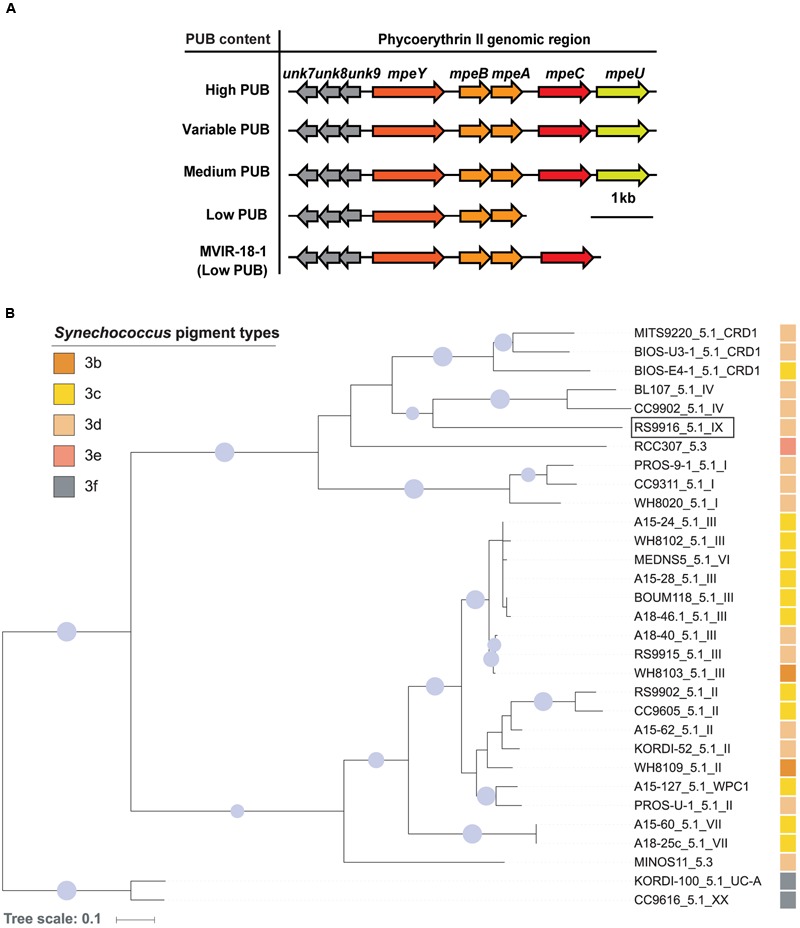
**Genomic context and phylogeny of *mpeU*. (A)** Genomic comparison of the phycoerythrin II genomic region, ordered by PUB content. **(B)** Maximum likelihood phylogenetic tree of MpeU. Sequence names include *Synechococcus* strain names, subcluster and clades (e.g., RS9916_5.1_IX), as defined in previous studies (Scanlan et al., 2009; Farrant et al., 2016). The pigment type of each strain is indicated by a colored square. The pigment type of the two strains at the root of the tree was not formally described (3f) but is likely to be high PUB (Xia et al., 2017). Only bootstrap values higher than 70% are shown by circles at nodes, and their size is proportional to bootstrap values. The RS9916 strain used in the present study is indicated by a rectangle.

Bioinformatic analyses of MpeU using Phyre^2^ (Kelley and Sternberg, 2009) demonstrated that its predicted structure has an alpha helical conformation, and contains a PBS lyase HEAT-like domain (Supplementary Figure S1), as previously reported for several characterized phycobilin lyases of the CpcE/F family (Storf et al., 2001; Blot et al., 2009; Shukla et al., 2012). From comparative genomic analyses of 54 sequenced marine *Synechococcus* and *Cyanobium* strains exhibiting a variety of pigment types (Supplementary Table S3), we found that *mpeU* is present in all 29 strains that have a medium (3b), high (3c) and variable PUB (3d) content, while it is absent in all strains that either have a low (3a) PUB content or completely lack PUB. All *Synechococcus* MpeU proteins display a high degree of similarity (Supplementary Figure S2) and have similar predicted structures using Phyre^2^. The combination of structural information and phyletic pattern led us to hypothesize that MpeU is a PEB lyase-isomerase, i.e., an enzyme that binds a PEB chromophore and transforms it into PUB by isomerization, like RpcG or MpeZ (Blot et al., 2009; Shukla et al., 2012). Interestingly, the *mpeU* gene is always located within a specific genomic region (**Figure 1A**), which contains several genes encoding proteins involved in the biosynthesis of PEII. This includes the PE-II α- and β-subunits (MpeA and MpeB), a PEII-specific linker polypeptide (MpeC; Six et al., 2005), another putative lyase (MpeY) and three conserved hypothetical proteins (Unk7, 8, and 9). This particular genomic context suggests that suggests that MpeU is a lyase-isomerase that specifically acts on PEII.

Phylogenetic analyses of MpeU show that this protein does not follow the phylogeny of vertically inherited genetic markers, such as the 16S rRNA gene (Scanlan et al., 2009), or *petB* (Farrant et al., 2016), suggesting that it has been laterally transferred between lineages during the evolution of the *Synechococcus* genus (**Figure 1B**; Supplementary Figure S3), as previously shown for other PEII genes (Six et al., 2007). For instance, although closely related, the two subcluster 5.3 strains RCC307 and MINOS11 do not group together in the MpeU tree. Similarly, clade VI strain MEDNS5, which with the *petB* marker groups with clades VII and CRD1, falls within clade III strains using the MpeU protein. It is also noteworthy that all low PUB strains lack both *mpeC* and *mpeU*, except MVIR-18-1which only lacks *mpeU* (**Figure 1A**).

### *mpeU* Mutant Cells have Decreased PUB Fluorescence

**FIGURE 2:**
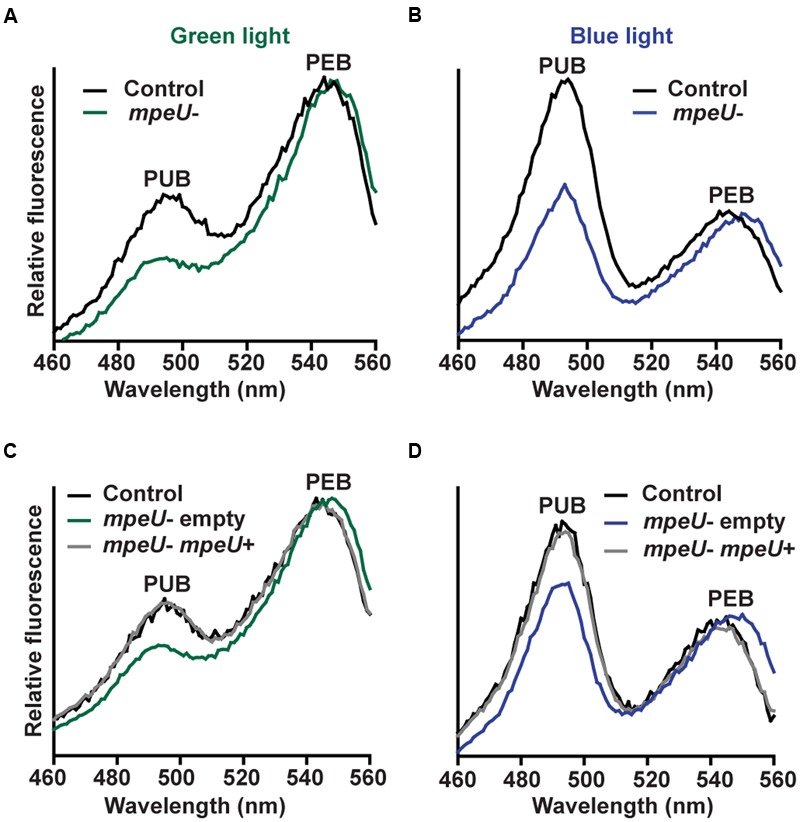
**The *mpeU* interruption mutant has a decreased Ex_495_:Ex_545_ ratio. (A,B)** Fluorescence excitation spectra, with emission set at 580 nm, for the *mpeU* mutant (green and blue lines) and control cells (black lines). **(C,D)** Fluorescence excitation spectra of the *mpeU* mutant cells with empty vector (green and blue lines), control cells (black lines) and the *mpeU* mutant cells with a vector expressing *mpeU* (gray lines) in green light **(C)** or blue light **(D)**. All spectra shown are an average of three independent replicates.

To test the hypothesis that MpeU is required for high PUB content, we made an *mpeU* interruption mutant in the model chromatically acclimating (3d) strain 9916 (Supplementary Figure S4). The spectral phenotype of the *mpeU* mutant was then compared to control cells by fluorescence excitation spectroscopy (**Figures 2A,B**). Consistent with our hypothesis, *mpeU* mutant cells had a lower PUB:PEB ratio (Ex_495_:Ex_545_ ∼0.4 in GL and ∼1.1 in BL) than control cells (∼0.6 in GL and ∼1.6 in BL).

To complement the *mpeU* mutant phenotype, *mpeU* was cloned into an autonomously replicating plasmid and reintroduced, along with an empty vector as a control, into the *mpeU* mutant (Supplementary Figure S5). The *mpeU* gene alone completely complemented the mutant fluorescence excitation phenotype in GL (**Figure 2C**) and BL (**Figure 2D**), confirming that *mpeU* is required for wild-type PUB fluorescence.

When comparing the optical properties of the *mpeU* mutant to control cells, we noticed a small blue shift of the PUB fluorescence excitation peak and a red-shift in PEB fluorescence excitation peak (**Figures 2A–D**) as well as a red shift of the PE emission peak (Supplementary Figure S6). As expected, reintroduction of *mpeU* into the *mpeU* mutant was sufficient to eliminate these spectral shifts, likely due to improper chromophorylation of PEI or PEII (**Figures 2A–D**; Supplementary Figure S6).

### *mpeU* Mutant Cells have Decreased Phycobilisome Content

**FIGURE 3:**
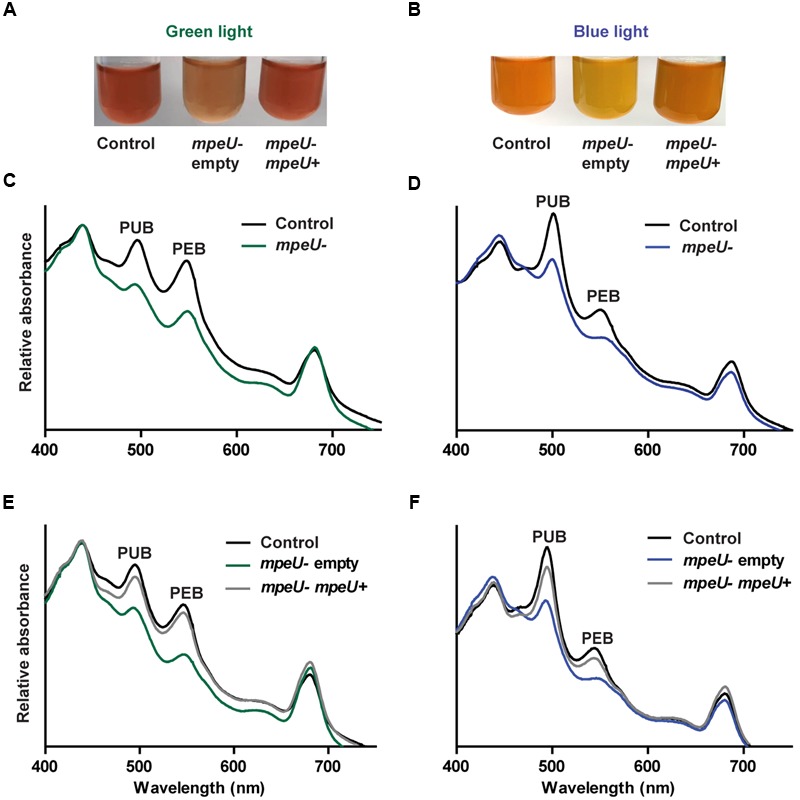
**Spectral phenotype of the *mpeU* mutant. (A,B)** Color phenotypes of control cell (left), *mpeU* mutant cells with empty vector (middle), and *mpeU* mutant cells expressing *mpeU* (right), in green light **(A)** and blue light **(B)**. The contrast was adjusted on some of the culture pictures in order to better view coloration of the cells. **(C,D)** Whole cell absorbance spectra for the *mpeU* mutant (green or blue lines) and control cells (black lines), in green light **(C)** and blue light **(D)**. **(E,F)** Whole cell absorbance spectra of *mpeU* mutant cells with empty vector (green or blue lines), *mpeU* mutant with vector expressing *mpeU* (gray lines), and control cells (black lines), in green light **(E)** and blue light **(F)**. All spectra shown are an average of three independent replicates.

The strong variations in PUB:PEB ratio occurring during CA4 causes WT 9916 cells to look pink in GL and orange in BL (Sanfilippo et al., 2016). However, *mpeU* mutant cells look yellow–orange in both BL and GL (**Figures 3A,B**), which is likely due to an overall lower phycobiliprotein content in the mutant. To examine the phycobiliprotein content of *mpeU* mutant cells, we used whole cell absorbance spectroscopy on *mpeU* mutant and control cells grown in GL and BL (**Figures 3C,D**). When normalized to chlorophyll absorbance, *mpeU* mutant cells had decreased absorbance from both PUB and PEB, suggesting that the loss of *mpeU* leads to a decreased overall PE content. The *mpeU* mutant phenotype in GL and BL was complemented by *mpeU* alone, confirming that mutation of *mpeU* is responsible for the altered phenotype (**Figures 3E,F**).

### *mpeU* is Required for Proper Phycobilisome Assembly

**FIGURE 4:**
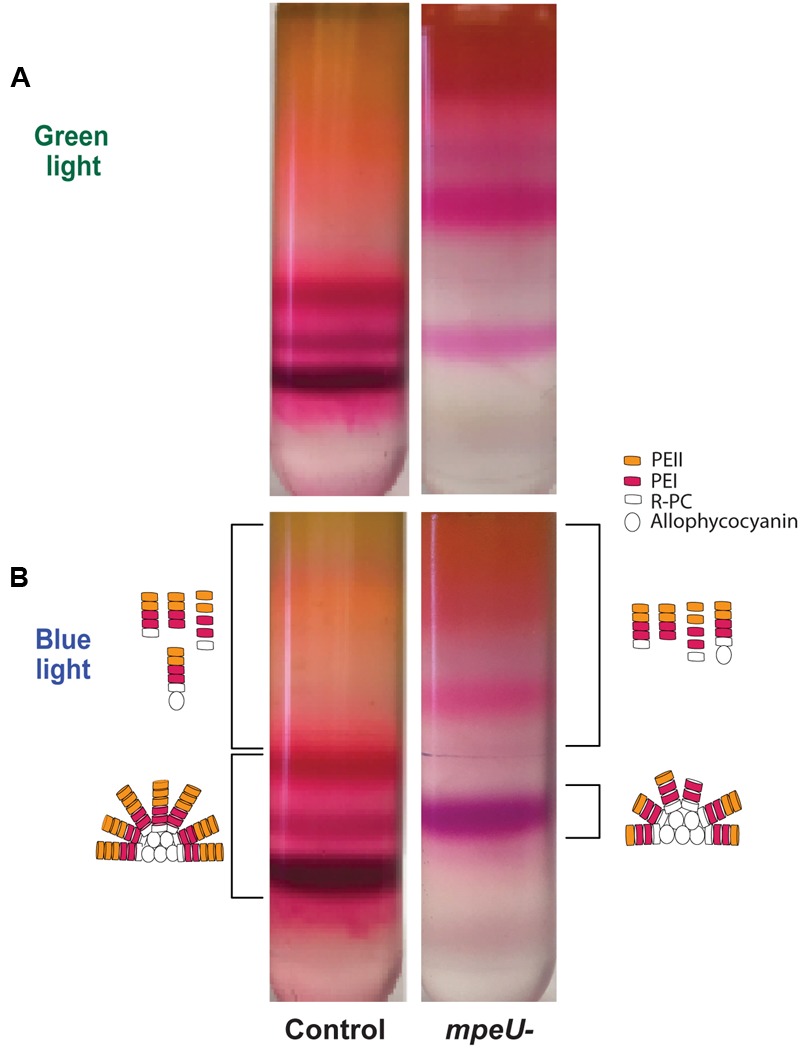
**Phycobilisomes (PBS) sucrose gradient separated for the *mpeU* mutant has a different banding pattern than control cells. (A)** green and **(B)** blue light. A cartoon representation of potential PBS or rod structures/components isolated from the bands are drawn to the side for blue light only. Bands collected are indicated by brackets. The color/shape for PEI, PEII, R-PC (R-Phycocyanin), and allophycocyanin are indicated.

Based on the decrease in overall PE content in *mpeU* mutant cells, we hypothesized that *mpeU* is required for proper biosynthesis of PEs and/or assembly of PBS. To test this hypothesis, we purified PBS from control and *mpeU* mutant cells using sucrose density gradient ultracentrifugation. After separating partially purified PBS on sucrose gradients, we observed that the PBS banding pattern was strikingly different in *mpeU* mutant cells compared to control cells (**Figure 4**). While most of the PBS from *mpeU* mutant cells were suspended in bands in the upper section of the gradient, PBS from control cells have three distinct bands in the lower section of the gradient. Because the densities of these complexes in the mutant are smaller than those seen in the WT, we conclude that the *mpeU* mutant cells have improperly assembled PBS, which likely causes the decrease in overall PBS content.

To further test whether or not the PBS of the *mpeU* mutant cells are properly assembled, we used fluorescence emission spectroscopy to measure energy transfer through purified PBS from different portions of the sucrose gradient. We measured fluorescence emission from 510 to 750 nm, with excitation set at 490 nm, for the different PBS fractions from *mpeU* mutant and control cells (Supplementary Figure S7). Consistent with the red shift observed in whole cells, there was a 2 nm red shift in GL (from 568 to 570 nm) and a 6 nm red shift in BL (565–571 nm) in the *mpeU* mutant when compared to the control spectrum. Also, we found primarily fluorescence emission from PE (at ∼570 nm) in the upper layer, little from PC (at ∼650 nm) and none from APC (at ∼675 nm), demonstrating that the upper bands of the gradient represented mostly uncoupled rod proteins in both the control and mutant (Supplementary Figures S7A,B).

In contrast, the lower bands of the gradient obtained from control cells exhibited strong PE, PC, and APC emission peaks, indicating that there was efficient energy transfer between the different phycobiliproteins and hence that these bands, especially the lowest and densest band of the gradient, consisted mostly of intact PBS (Supplementary Figures S7C,D). By comparison, the lowest band of the gradient from *mpeU* mutant cells had much lower relative PC and APC emission peaks (the latter was particularly reduced in GL) than control cells, suggesting that the PE in the PBS of this bottom band was not efficiently transferring energy to PC and APC. This may be due to lower PE content in rods of the *mpeU* mutant and/or to improper chromophorylation of PE that may disrupt energy transfer. From these results, we conclude that MpeU is required for the proper assembly of PE within the PBS in 9916.

### *mpeU* Mutant Cultures have a More Pronounced Growth Defect in Blue than Green Light

**FIGURE 5:**
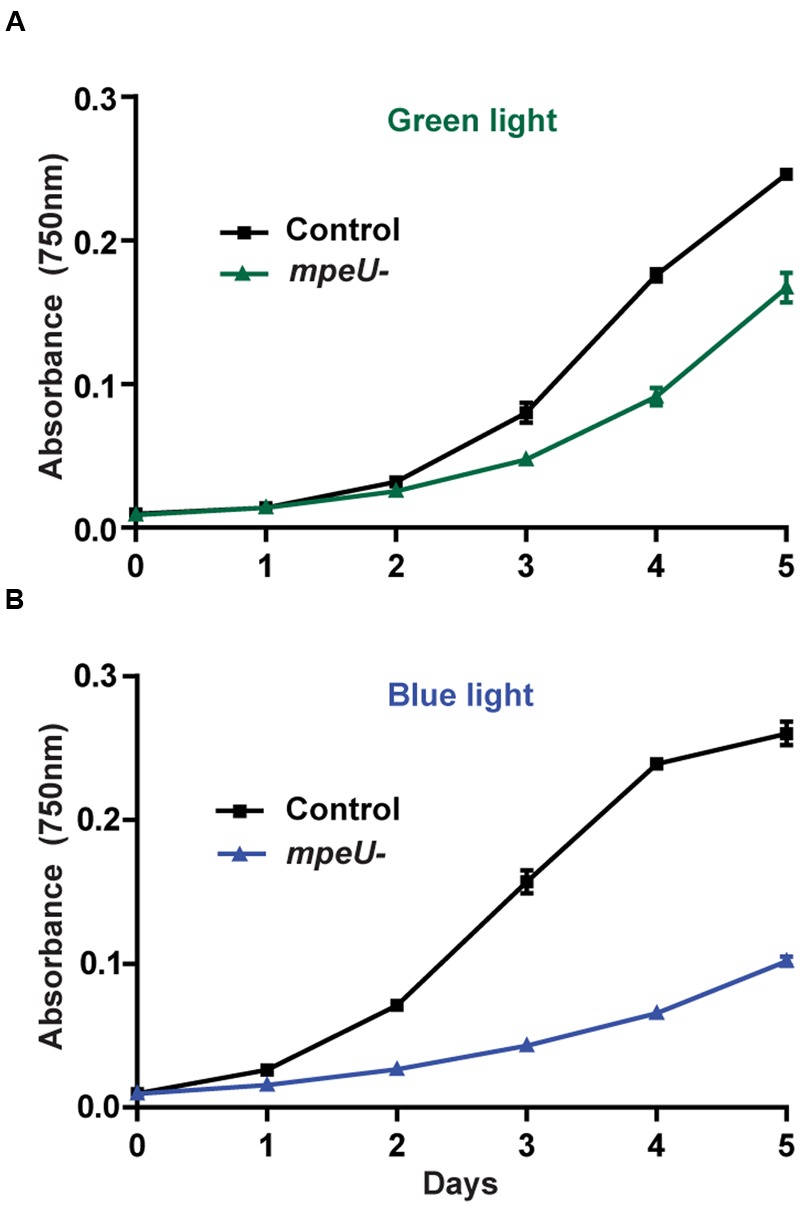
**The *mpeU* mutant growth defect is larger in blue light than in green light.** Cell density, measured by absorbance at 750 nm, during growth in green light **(A)** and blue light **(B)** for control cells (black lines) and *mpeU* mutant cells (green or blue lines). Error bars show SEM for three independent replicates.

Given the PBS assembly defect occurring in the *mpeU* mutant, we hypothesized that *mpeU* mutant cells may have decreased growth compared to control cells. Comparative growth experiments showed that *mpeU* mutant cells had decreased growth compared to control cells in both GL and BL, but the growth defect was more pronounced in BL (**Figure 5**), which is consistent with MpeU having a role in PUB attachment and absorption of BL. Therefore, we conclude that MpeU is not only required for proper PBS content and assembly, but it is also required to provide optimal growth in marine environments rich in BL.

## Discussion

Our long-term goal is to understand how marine *Synechococcus*, the world’s second most abundant group of photosynthetic phytoplankton, thrives in environments specifically enriched in either blue or green light. Green light predominates in the upper layer of coastal and nutrient-rich waters, while blue light predominates at the bottom of the euphotic zone and in offshore, nutrient-poor waters (Kirk, 1994).

Using comparative genomics and a refined physiological and biochemical characterization of mutants, we previously unveiled the function of MpeZ, a lyase-isomerase specifically involved in CA4 (Shukla et al., 2012). In the present study, we focused on MpeU, which we found to be present in all strains that have medium, high or variable PUB content and absent in all strains with low PUB content (Supplementary Table S3). MpeU is encoded in strains with medium PUB content (3b), such as WH8103 or WH8109, which possess the complete genetic equipment of CA4-capable (3d) strains but are blocked in the green light phenotype, likely because their CA4 regulatory machinery is impaired (Humily et al., 2013). When *mpeU* was inactivated in 9916, cells exhibited an Ex_495_:Ex_545_ ratio of ∼0.4, similar to low PUB (3a) strains, such as WH7803, but also to *Synechococcus* isolates naturally lacking *mpeU*, such as MVIR-18-1, which otherwise has the same PBS gene content as 3d strains (Humily et al., 2013). When *mpeU* was reintroduced on a plasmid, 9916 cells were restored to WT PUB levels (**Figure 3**). Taken together, these data suggest that *mpeU* is one of the genes required for PUB synthesis and attachment on phycoerythrin and drives BL adaptation throughout the *Synechococcus* genus.

The coloration and whole cell absorption spectra indicate that *mpeU* mutant cells are producing fewer phycobiliproteins, and especially less PEI and PEII than control cells. The sequence of MpeU is predicted to be structurally similar to other phycobilin lyases in the CpcE/F family such as MpeZ (Supplementary Figure S1; see also http://cyanolyase.genouest.org/), suggesting that its role in BL acclimation is to attach PEB at a particular Cys on a PEI and/or PEII subunit and to isomerize it to PUB. The particular genomic context of *mpeU* further suggests a specificity for PEII. The phenotype of the *mpeU* mutant is consistent with phenotypes measured for other bilin lyase mutants. In *Synechococcus* sp. PCC 7002, *cpcE* and *cpcF* mutants synthesized much less PC than the WT, which caused the cells to appear yellowish-green and resulted in less dense PBS produced, as measured by slower migration in sucrose density gradients (Swanson et al., 1991; Zhou et al., 1992). PC synthesized in those mutants was missing a PCB chromophore on the α-subunit. Similarly, characterization of the *cpcS* and *cpcU* lyase mutants in *Synechococcus* sp. PCC 7002 showed that the PC β-subunit was missing a chromophore, and mass spectrometry analyses revealed that CpcB from mutants contained some non-covalently bound PCB (Shen et al., 2008). In *pecE*/*pecF* mutants, PCB (rather than the native phycoviolobilin or PVB) was shown to be attached to PecA, providing the first evidence that PecE and PecF together formed a heterodimeric PCB lyase-isomerase (Jung et al., 1995). It was not clear whether in the *pecE*/*pecF* mutants, PCB associated with PecA and slowly became auto-ligated to the Cys or whether another lyase was able to ligate this chromophore with reduced efficiency. In site-directed mutants of *cpcB* or *apcE*, where the Cys bilin attachment site of the encoded protein was changed to Ala or Ser, PCB was bound non-covalently, and the spectrum of the phycobiliprotein was red-shifted due to the extra double bond at ring A that lengthens the conjugated double bond system (Zhou et al., 1992; Debreczeny et al., 1993; Anderson and Toole, 1998). Such a red-shifted chromophore phenotype was also observed in our *mpeU* mutant in fluorescence scans of whole cells and of purified PBS (Supplementary Figures S6 and S7). If MpeU is, as we hypothesize, a PEB lyase-isomerase for PEII (or PEI or both PEI and PEII), then in its absence, it is possible either that PEB slowly auto-ligates itself to the Cys to form the thioether linkage or that another lyase may be able to ligate PEB to this site. If it occurs at all, this alternative PEB binding process in the *mpeU* mutant is probably not very efficient, since cells display a low PBS content (**Figure 3**). The observed red-shift could be due to PEB (covalently or non-covalently) occurring where PUB should be in the WT PBS, or to the complete lack of chromophore at the affected Cys.

Because there is a phenotype of the *mpeU* mutant in both GL and BL and the defect is much more pronounced in BL, we suspect that MpeU is likely a PEB lyase-isomerase that is responsible for adding PUB. There are several possible PUB attachment sites within the rod PBS of 9916 in BL conditions besides the previously characterized MpeZ-specific site on MpeA-Cys83 (Shukla et al., 2012) and RpcG-specific site on RpcA-Cys84 (Blot et al., 2009): MpeA-Cys75; MpeA-Cys140; CpeA-Cys139; CpeB-Cys50/61; MpeB-Cys50/61; and MpeC-Cys49. Alternatively, since no lyases implicated in the chromophorylation of PEII linker polypeptides (such as MpeC or MpeD; Wilbanks and Glazer, 1993; Six et al., 2005) have been characterized thus far, MpeU might be a lyase for these linkers. However, ApcE, the large core-membrane linker protein, was shown to be capable of auto-ligating its PCB chromophore (Zhao et al., 2005; Biswas et al., 2010) and a similar mechanism could also occur for PEII linker polypeptides. Even though the site specificity of MpeU remains to be determined, our data clearly support a function of MpeU as a PEB lyase-isomerase responsible for PUB attachment on PEI or, more likely, PEII. MpeU is therefore a critical enzyme for adaptation of marine *Synechococcus* to environments where blue light predominates, which is the case in vast zones of the world’s ocean, notably in the central oceanic gyres (Kirk, 1994).

## Author Contributions

RM, JES, AN, and JAS contributed to the design of the work, performed experiments and data analysis, and participated in the drafts and revisions. NAE, FP, and LG performed data analysis and participated in the drafts and revisions. DK and WS made substantial contributions in the conceptual design of the work, in the data analysis, and in the writing and revisions of this manuscript.

## Conflict of Interest Statement

The authors declare that the research was conducted in the absence of any commercial or financial relationships that could be construed as a potential conflict of interest.

## Footnotes

1 http://roscoff-culture-collection.org/

2 http://www.sb-roscoff.fr/Phyto/collect.html

3 http://manatee.sourceforge.net/igs/index.shtml

4 http://orthomcl.org/orthomcl/

5 www.sb-roscoff.fr/cyanorak/

6 http://itol.embl.de
