# Supplementary material for: Adaptation to Blue Light in Marine Synechococcus Requires MpeU, an Enzyme with Similarity to Phycoerythrobilin Lyase Isomerases
Source: Front Microbiol. 2017 Feb 21;8:243. doi: 10.3389/fmicb.2017.00243 (PMC5318389; doi:10.3389/fmicb.2017.00243)
Supplement: Supplementary file 1 [file Image_1.pdf]

Supplementary Material

Adaptation to blue light in marine *Synechococcus* requires MpeU, an enzyme with similarity to phycoerythrobilin lyase isomerases

*Rania M. Mahmoud[a,b], *Joseph E. Sanfilippo[a], Adam A. Nguyen[c,d], Johann A. Strnat[a], Frédéric Partensky[e], Laurence Garczarek[e], Nabil Abo El Kassem[b], David M. Kehoe[a,f] and Wendy M. Schluchter[c,d,1]

[a]Department of Biology, Indiana University, Bloomington, Indiana, 47405 U.S.A.; [b] Department of Botany, Faculty of Science, University of Fayoum, 63514 Fayoum, Egypt ;[c]Department of Biological Sciences, University of New Orleans, New Orleans, LA 70148; [d]Department of Chemistry, University of New Orleans, New Orleans, LA 70148; [e]Sorbonne Universités, Université Pierre et Marie Curie University Paris 06, CNRS, UMR 7144, Station Biologique, Plankton Group, 29688 Roscoff, France; [f]Indiana Molecular Biology Institute, Indiana University, Bloomington, Indiana, 47405 U.S.A.

*These authors contributed equally to this work

[1]To whom correspondence should be addressed. Department of Biological Sciences
232 Biology Building
University of New Orleans
New Orleans, LA 70148 U.S.A.
E-mail: wschluch@uno.edu
Tel: (504) 280-7194

1. Supplementary Data

Figure S1. Structural analysis of MpeU using Phyre2 (Kelley and Sternberg, 2009)**.** (A) A total of 241 residues (80%) were modeled with 99.9% confidence to the PBS lyase HEAT-like repeat family. (B) Predicted 3-D model of MpeU. Alignment of MpeU and the PBS lyase HEAT-like repeat and their predicted secondary structure (C).

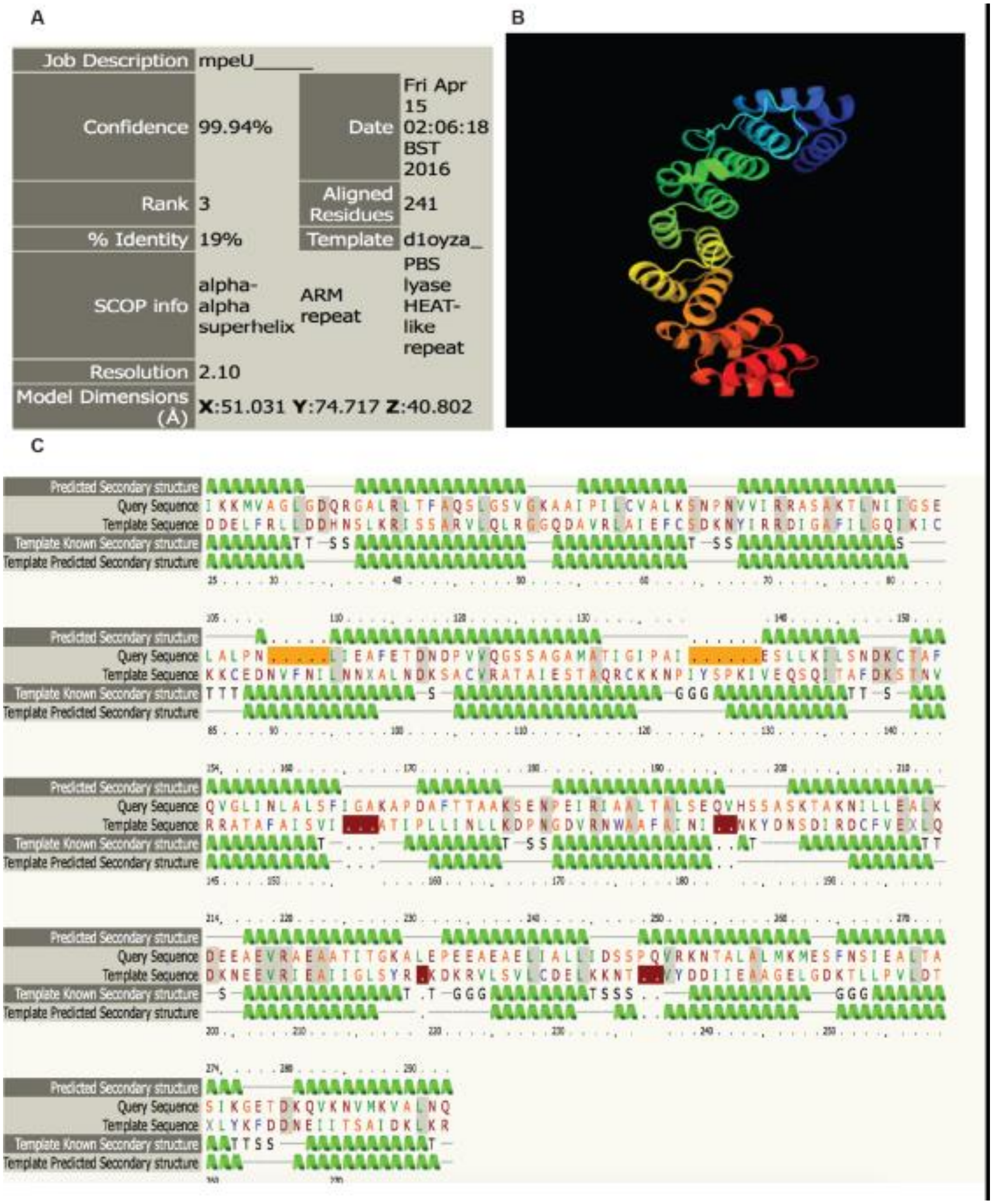

Figure S2. Multiple alignment of a selection of MpeU sequences performed with ClustalW2 (Sievers and Higgins, 2014). Sequence names include *Synechococcus* strain names, subcluster, clades and pigment type (e.g., RS9916_5.1_IX_3d), as defined in previous studies (Scanlan et al., 2009; Farrant et al., 2016). Characters highlighted in black or gray represent identical or similar residues, respectively.

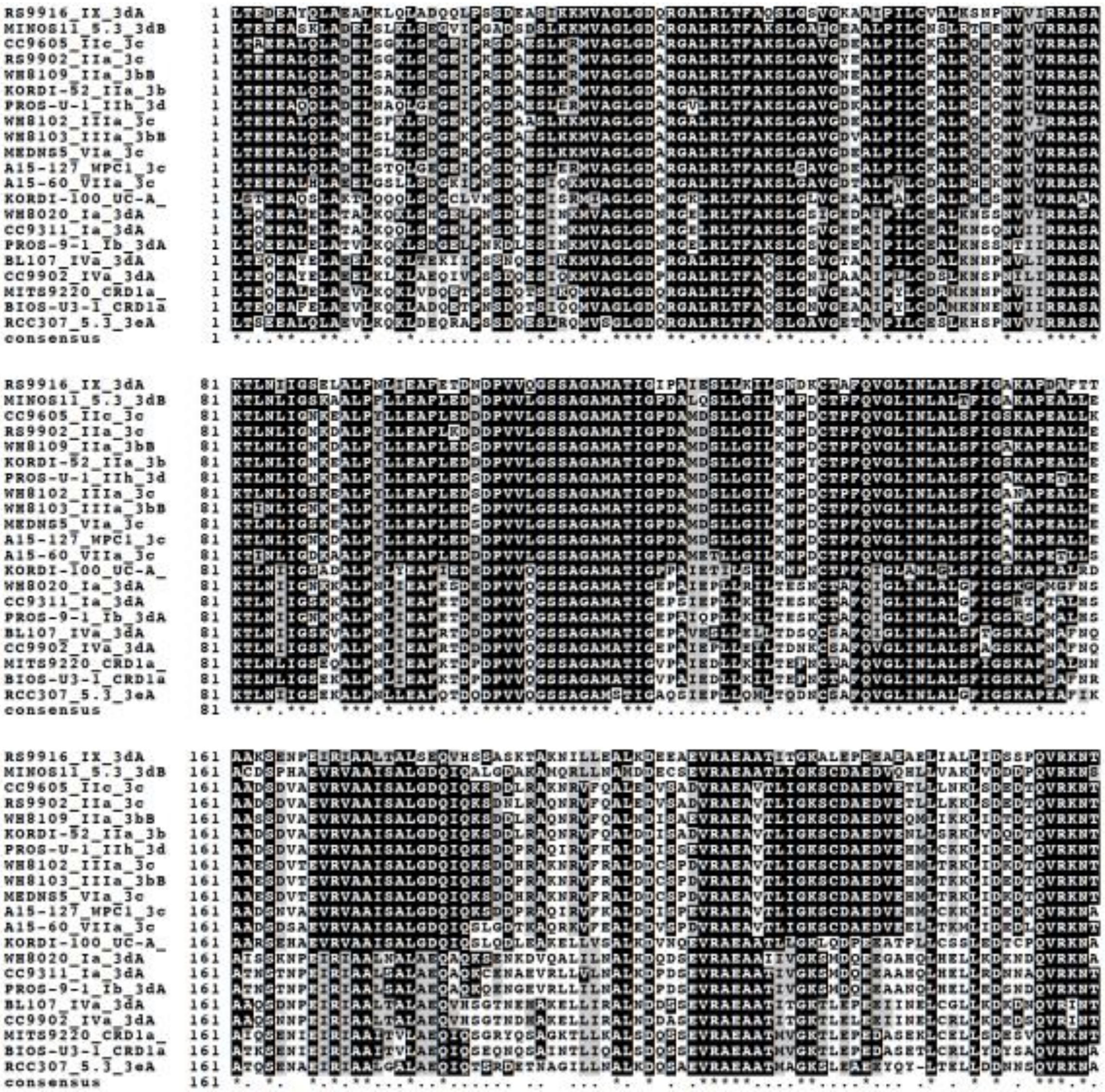

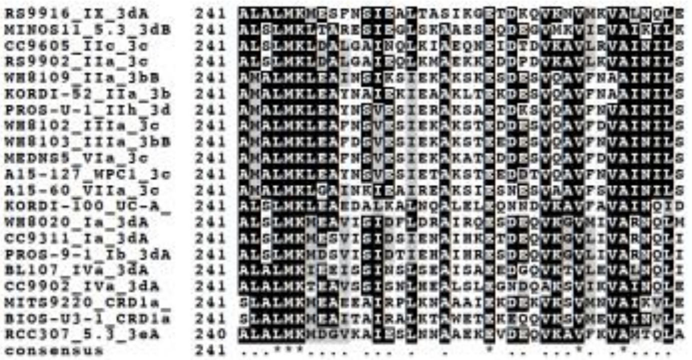
RS9916_IX_3dA 241
MINOS11_5.3_3dB 241
CC9605_IIc_3c 241
RS9902_IIa_3c 241
WH8109_IIa_3bB 241
KORDI-52_IIa_3b 241
PROS-U-1_IIh_3d 241
WH8102_IIIa_3c 241
WH8103_IIIa_3bB 241
MEDNS5_VIa_3c 241
A15-127_WPC1_3c 241
A15-60_VIIa_3c 241
KORDI-100_UC-A_ 241
WH8020_Ia_3dA 241
CC9311_Ia_3dA 241
PROS-9-1_Ib_3dA 241
BL107_IVa_3dA 241
CC9902_IVa_3dA 241
MITS9220_CRD1a_ 241
BIOS-U3-1_CRD1a 241
RCC307_5.3_3eA 240
consensus 241

Figure S3. Comparison of the phylogenetic maximum likelihood trees obtained with the vertically inherited marker gene *petB*, encoding cytochrome b_6 (Farrant et al., 2016), and with the putative lyase MpeU. Strain description include *Synechococcus* strain names, subcluster, clades and pigment type (e.g., RS9916_5.1_IX_3d), as defined in previous studies (Scanlan et al., 2009; Farrant et al., 2016). Each clade is coded with a different color.

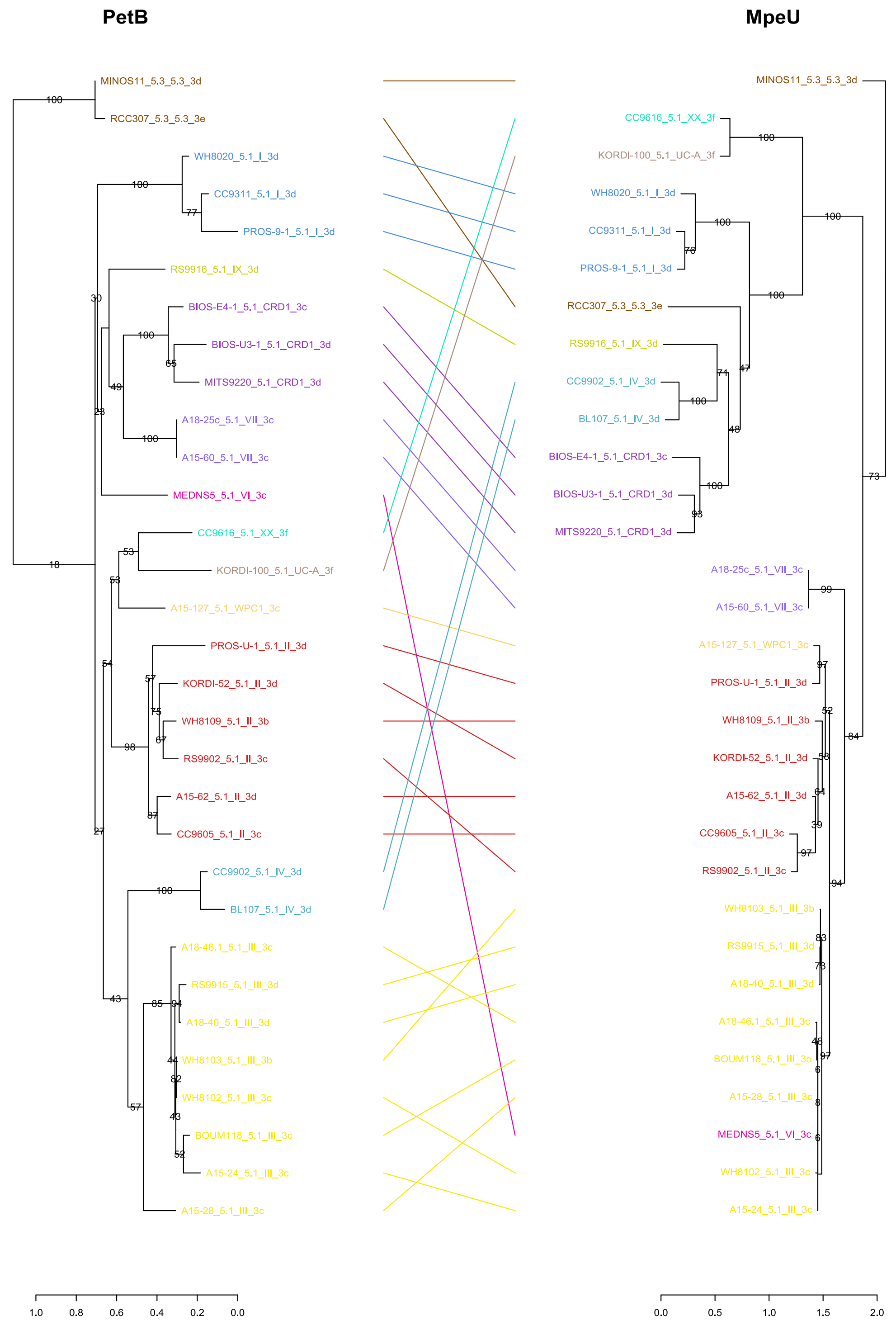

Figure S4. *mpeU* interruption construct and verification. (A) *mpeU* gene map in RS9916, (B) gene map of the *mpeU* interruption mutant and agarose gel verification with three independent replicates of *mpeU* mutant and wild type using two different PCR amplification approaches for mutant confirmation. (C and D) Restriction maps and DNA Southern blot analysis showing *mpeU*-containing DNA fragments in WT and the *mpeU* mutant. (C) Restriction map of WT RS9916 at *mpeU* location. (D) Restriction map of *mpeU* mutant at the same location and Southern Blot analysis of WT and *mpeU* mutant DNA digested with either NheI or SacI.

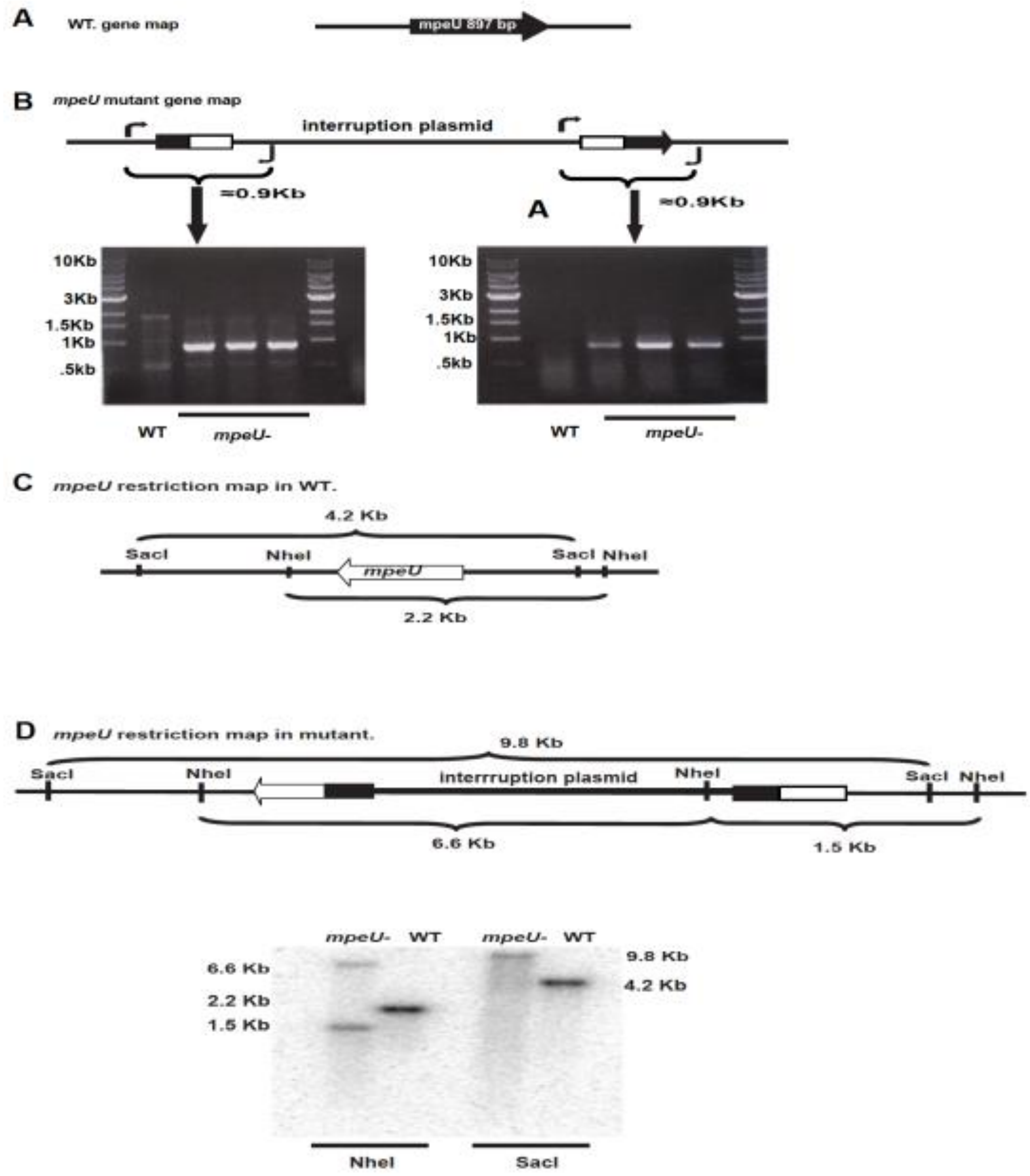

Figure S5. (A) Plasmid construct used for *mpeU* complementation, (B) PCR agarose gel for *mpeU* mutant complementation verification along with wild type control and the empty vector of the autonomously replicated plasmid.

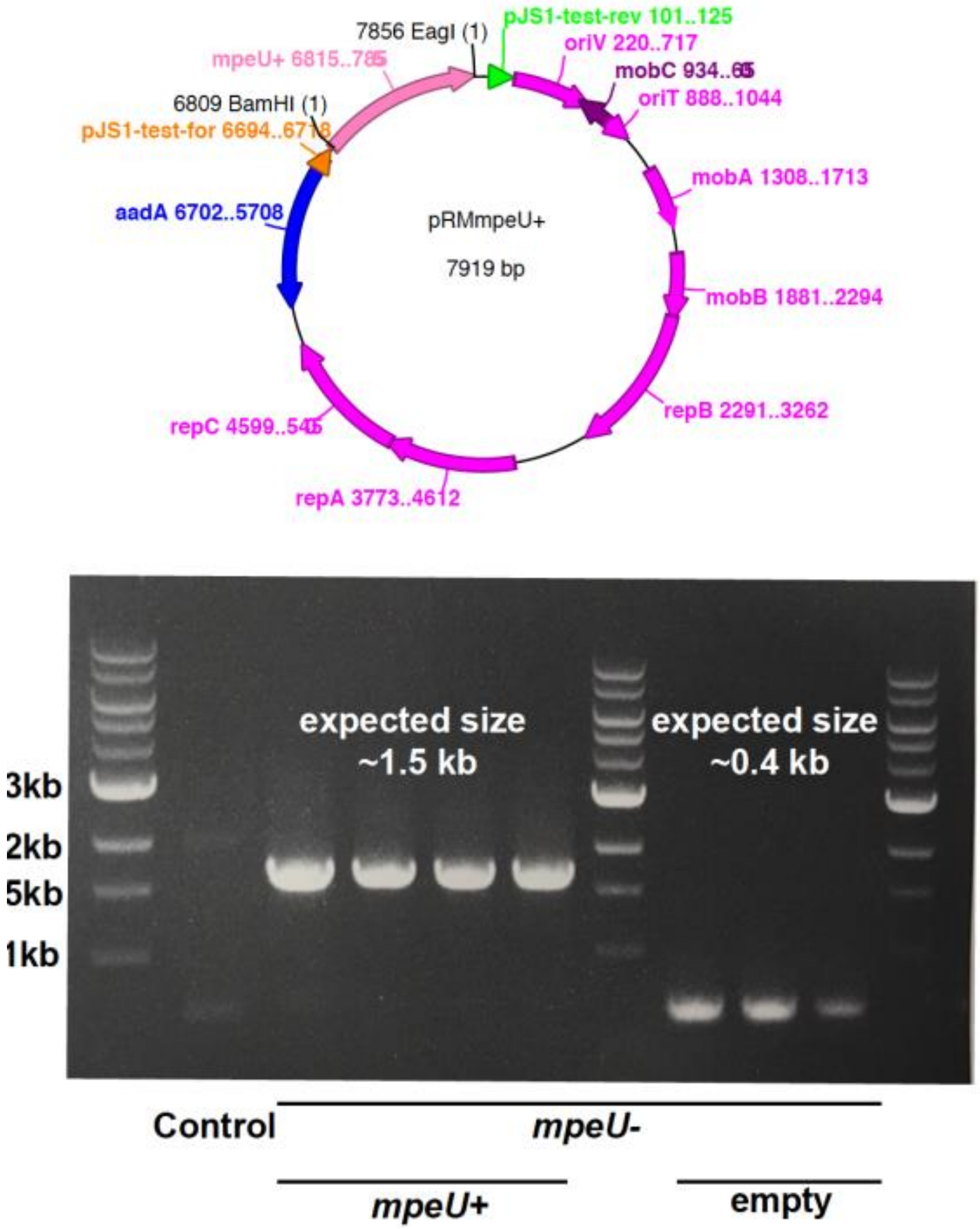

Figure S6. Complementation of *mpeU* mutant fluorescence emission peak shifts. (A) Fluorescence scan with emission set at 580nm focused on the PEB peak in green light, (B) Fluorescence scan with emission set at 580nm focused on the PEB peak in blue light, for control cells (black lines), *mpeU* mutant with empty vector (grey lines) and *mpeU* mutant with *mpeU* gene expressed on the same vector (green or blue lines).

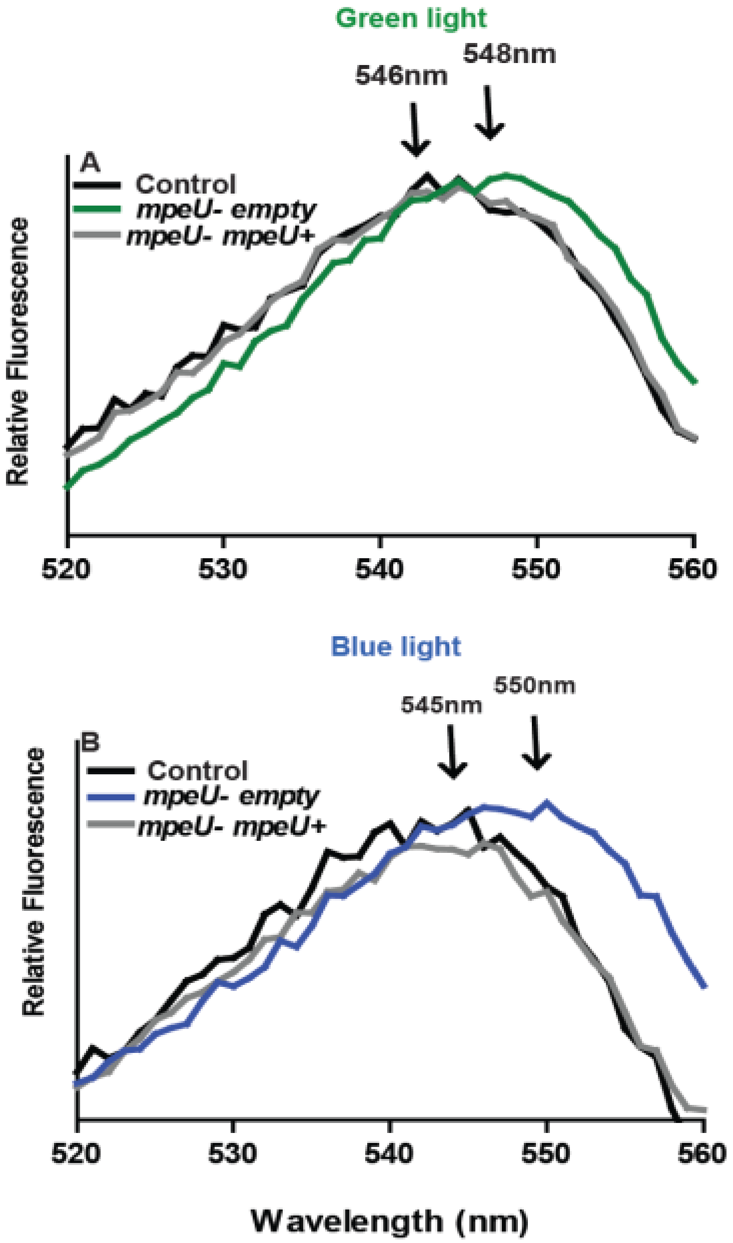

Figure S7. Fluorescence from top and bottom bands of sucrose gradient separated PBS are different in control and *mpeU* cells. (A, B) Fluorescence spectroscopy with excitation set at 490 nm and emission from 510 nm to 700 nm for purified phycobilisomes isolated from the top bands; (C, D) bottom bands of the sucrose gradients. (A, C) in green light and (B and D) blue light for *mpeU* (green and blue lines) and control cells (black lines).

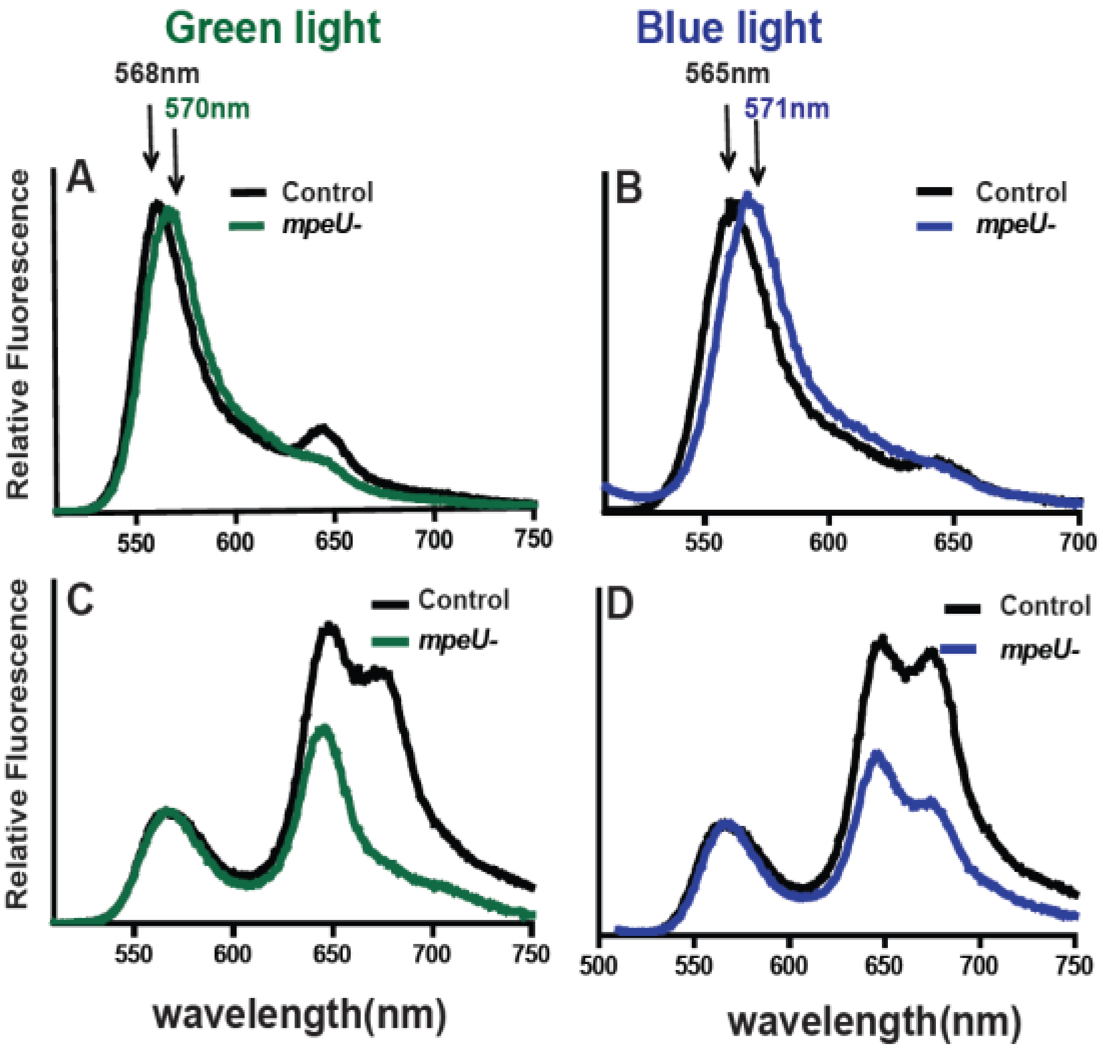

Table S1. List of primers used in this study. The restriction enzyme sites are underlined.

Primer name	Sequence 5'- 3'
Int-BamHI-mpeU-for	ACT<u>GGATCC</u>CAGCTCAGATGAGGCCTCGATCAA
Int-BamHI-mpeU-rev	ACT<u>GGATCC</u>GCTTCTAGTAAAATATTTTTTGCGGTTTTGCTCGC
mpeU-probe-for	GCTCAGTAGGTAAAGCAGCGATCC
mpeU-probe-rev	GGTTTTGCTCGCACTGGAATGGA
Comp-BamHI-mpeU-for	GCA<u>GGATCC</u>TTTGACAGAAGTCGAATAACTCCTCAAAGGT
Comp-EagI-mpeU-rev	ATA<u>CGGCCG</u>TTATGAATTTATTTCTAGCTGGTTGAGTGCCAC

Table S2. List of plasmids used in this study.

Plasmid name	Resistance	Description
pMUT100	Kan (50 μg ml^{-1})	Used to clone *mpeU* insert for interruption
pJASmpeU	Kan (50 μg ml^{-1})	Used to interrupt *mpeU*
pJS1	Spec (20 μg ml^{-1})	Empty vector, used for complementation
pJS1-mpeU	Spec (20 μg ml^{-1})	Used to complement *mpeU* mutant

Table S3. Presence/absence of the *mpeU* gene in 54 sequenced marine *Synechococcus* or *Cyanobium* strains. Strains are ordered by phycobiliprotein composition of their PBS rods and their relative content in PUB, two characteristics used to define their respective pigment types (Six et al., 2007; Humily et al., 2013).

PBS rods	PUB content	Strain	Pigment type	*mpeU*
PC+PEI+PEII	Variable PUB	BIOS-U3-1	3d	Yes
		BL107	3d	
		CC9311	3d	
		CC9902	3d	
		MITS9220	3d	
		PROS-9-1	3d	
		RS9916	3d	
		WH8020	3d	
		A15-62	3d	
		A18-40	3d	
		MINOS11	3d	
		PROS-U-1	3d	
		RS9915	3d	
		RCC307	3e	
	High PUB	A15-127	3c	Yes
		A15-24	3c	
		A15-28	3c	
		A15-60	3c	
		A18-25c	3c	
		A18-46.1	3c	
		BOUM118	3c	
		CC9605	3c	
		MEDNS5	3c	
		RS9902	3c	
		WH8102	3c	
		BIOS-E4-1	3c	
		CC9616	3f	
		KORDI-100	3f	
	Medium PUB	KORDI-52	3b	Yes
		WH8103	3b	
		WH8109	3b	
	Low PUB	M16.1	3a	No
		NOUM97013	3a	
		ROS8604	3a	
		RS9907	3a	
		SYN20	3a	
		TAK9802	3a	
		WH7803	3a	
		KORDI-49	3a	
		MVIR-18-1	3a	
		WH8016	3a	
PC+PEI	No PUB	A15-44	2	No
		BMK-MC-1	2	
		CB0205	2	
		PROS-7-1	2	
		WH7805	2	
PC	No PUB	CB0101	1	No
		NS01	1	
		PCC6307	1	
		PCC7001	1	
		RS9909	1	
		RS9917	1	
		WH5701	1	
		WH8101	1	

Supplementary References:

Farrant, G.K., Dore, H., Cornejo-Castillo, F.M., Partensky, F., Ratin, M., Ostrowski, M., Pitt, F.D., Wincker, P., Scanlan, D.J., Iudicone, D., Acinas, S.G., and Garczarek, L. (2016). Delineating ecologically significant taxonomic units from global patterns of marine picocyanobacteria. *Proc Natl Acad Sci U S A* 113**,** E3365-3374. doi:10.1073/pnas.1524865113

Humily, F., Partensky, F., Six, C., Farrant, G.K., Ratin, M., Marie, D., and Garczarek, L. (2013). A gene island with two possible configurations is involved in chromatic acclimation in marine *Synechococcus*. *PLoS One* 8**,** e84459. doi:10.1371/journal.pone.0084459

Kelley, L.A., and Sternberg, M.J. (2009). Protein structure prediction on the Web: a case study using the Phyre server. *Nat Protoc* 4**,** 363-371. doi:10.1038/nprot.2009.2

Scanlan, D.J., Ostrowski, M., Mazard, S., Dufresne, A., Garczarek, L., Hess, W.R., Post, A.F., Hagemann, M., Paulsen, I., and Partensky, F. (2009). Ecological genomics of marine picocyanobacteria. *Microbiol Mol Biol Rev* 73**,** 249-299. doi:10.1128/MMBR.00035-08

Sievers, F., and Higgins, D.G. (2014). Clustal omega. *Curr Protoc Bioinformatics* 48**,** 3 13 11-16. doi:10.1002/0471250953.bi0313s48

Six, C., Thomas, J.C., Garczarek, L., Ostrowski, M., Dufresne, A., Blot, N., Scanlan, D.J., and Partensky, F. (2007). Diversity and evolution of phycobilisomes in marine *Synechococcus* spp.: a comparative genomics study. *Genome Biol* 8**,** R259. doi:10.1186/gb-2007-8-12-r259
